# Exploring recent advances in signaling pathways and hallmarks of uveal melanoma: a comprehensive review

**DOI:** 10.37349/etat.2025.1002306

**Published:** 2025-04-02

**Authors:** Majid Banimohammad, Parsa Khalafi, Danial Gholamin, Zahra Bangaleh, Nahid Akhtar, Abhishikt David Solomon, Pranav Kumar Prabhakar, Samira Sanami, Ajit Prakash, Hamidreza Pazoki-Toroudi

**Affiliations:** IRCCS Istituto Romagnolo per lo Studio dei Tumori (IRST) “Dino Amadori”, Italy; ^1^Physiology Research Center, Iran University of Medical Sciences, Tehran 1449614535, Iran; ^2^Department of Medical Nanotechnology, School of Advanced Technologies in Medicine, Tehran University of Medical Sciences, Tehran 1417613151, Iran; ^3^School of Bioengineering and Biosciences, Lovely Professional University, Phagwara 144411, India; ^4^Adams School of Dentistry, Oral and Craniofacial Biomedicine, University of North Carolina, Chapel Hill, NC 27599, USA; ^5^School of Allied Medical Sciences, Lovely Professional University, Phagwara 144411, India; ^6^Parul Institute of Applied Sciences & Research and Development Cell, Parul University, Vadodara 391760, India; ^7^Abnormal Uterine Bleeding Research Center, Semnan University of Medical Sciences, Semnan 3514799442, Iran; ^8^Department of Biochemistry and Biophysics, University of North Carolina, Chapel Hill, NC 27599, USA; ^9^Department of Physiology, School of Medicine, Iran University of Medical Sciences, Tehran 1449614535, Iran

**Keywords:** Uveal melanoma, rat sarcoma virus (RAS) pathway, Akt pathway, Hippo pathway, retinoblastoma (Rb) pathway, p53 pathway, hallmarks of cancer

## Abstract

The purpose of this review was to provide a comprehensive review of the latest insights on the pathogenesis of uveal melanoma (UM) and its intracellular pathways. This article covers the epidemiology of UM, racial predispositions, cytogenetic and chromosomal alterations, gene mutations, key defective pathways, and their underlying mechanisms, as well as the application of hallmarks of cancer to UM. A key knowledge gap remains in identifying the most effective targeted therapy and determining the central pathway linking multiple signaling networks. UM is a malignant tumor arising from uveal melanocytes, predominantly affecting the choroid, with both genetic and epigenetic contributors. Key cytogenetic alterations include monosomy 3, chromosome 6p gain, chromosome 1p loss, and chromosome 8q gain. The most important UM-related signaling pathways are RAS/MAPK, PI3K/Akt/mTOR, Hippo-YAP, retinoblastoma (Rb), and p53 pathways. In the RAS/MAPK pathway, *GNAQ*/*GNA11* mutations occur which account for more than 80% of UM cases. The PI3K/Akt/mTOR pathway promotes cyclin D1 overexpression and MDM2 upregulation, leading to p53 pathway inhibition. *GNAQ*/*GNA11* mutations activate YAP via the Trio-RhoGTPase/RhoA/Rac1 signaling circuit in the Hippo-YAP pathway. Rb pathway dysregulation results from cyclin D1 overexpression or cyclin-dependent kinase inhibitor (CDKI) inactivation. In the p53 pathway, UM is characterized by *p53* mutations, MDM2 overexpression, and Bcl-2 deregulation. Eventually, the ARF-MDM2 axis serves as a critical link between the RAS and p53 pathways. Hallmarks of cancer, such as evasion of growth suppression and self-sufficiency in growth signals, are also evident in UM. Genetic and epigenetic alterations, including *NSB1*, *MDM2* and *CCND1* amplification, and *BAP1* mutations, play pivotal roles in UM pathobiology. Thus, UM exhibits a multifactorial pathology. By consolidating key mechanisms underlying UM pathogenesis, this review provides a comprehensive perspective on the involved pathways, offering insights that may facilitate the development of effective therapeutic strategies.

## Graphical abstract

**Graphical abstract.**
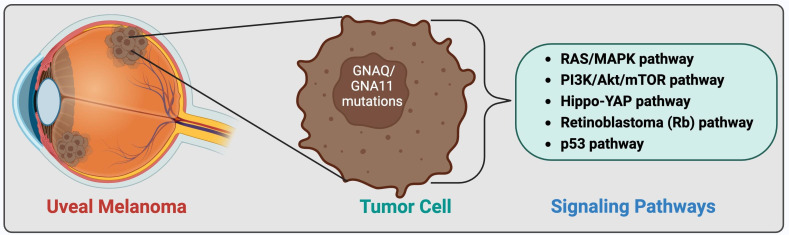
***GNAQ*/*GNA11* mutations affect several intracellular pathways that result in tumor cell growth and uveal melanoma formation.** *GNA11*: guanine nucleotide-binding protein subunit alpha-11; *GNAQ*: guanine nucleotide-binding protein G(q) subunit alpha; MAPK: mitogen-activated protein kinases; mTOR: mammalian target of rapamycin; PI3K: phosphoinositide 3 kinase; RAS: rat sarcoma virus; YAP: yes-activated protein. Created in BioRender. Prakash, A. (2025) https://BioRender.com/f79v771

## Introduction

Uveal melanoma (UM) is a rare type of cancer that develops in the uvea, comprising the iris, ciliary body, and choroid. UM is the most common primary intraocular cancer, arising from uveal melanocytes. UM is a malignant tumor that involves the choroid in 90% of cases, with the iris and ciliary body affected in the remaining 10% [1]. The incidence of UM is approximately five cases per million people in the US, with males exhibiting a 30% higher incidence than females. UM is predominantly found in the caucasian population. The median age at diagnosis of UM is estimated to be 62 years [2]. UM can lead to neovascular glaucoma, visual impairment, vision loss, pain, metastasis, and even death. Various therapeutic modalities have been developed for the treatment of intraocular tumors such as UM [2–4]. This review discusses the pathophysiology and intracellular pathways involved in UM.

UM, although rare, remains the most common primary intraocular malignancy. Effective primary treatments, such as radiation and enucleation, still exist; however, metastasis remains difficult to prevent or treat. Prognostic tools combining histopathological, molecular, and patient-specific markers enhance the prediction of metastatic risk. On the other hand, treatment of UM has advanced significantly, with immune checkpoint inhibitors and BRAF/MEK inhibitors forming the cornerstone of therapy. However, resistance to these treatments underscores the need for innovative approaches. Advances in monoclonal antibodies and small molecules have enabled the targeting of specific mediators driving the cancer phenotype. While the *BRAF^V600^* mutations in melanoma have guided effective therapies, other mutations, such as *NRAS* and *NF-1* loss-of-function mutations, remain difficult to target. Personalized medicine is increasingly focusing on the genetic landscape and molecular classifications to develop combination therapies [5, 6].

This comprehensive review presents the most recent information on the pathogenesis of UM and its intracellular pathways. It aims to summarize the major mechanisms of pathogenesis to provide a comprehensive understanding of the involved pathways, which could significantly aid in the design of treatment strategies.

## Pathobiology of UM

UM is characterized by malignant over-proliferation of ocular melanocytes, which are derived from melanoblasts. Melanocytes originate from non-pigmented melanoblasts, which arise from the neural crest and migrate during embryogenesis. These melanoblasts mature and differentiate into melanocytes within the uvea, or they generate melanocytic stem cells, which maintain ocular melanocytic functionality. The specific location of uveal melanocytic stem cells remains uncertain [7]. The differentiation of melanoblasts into melanocytes can be influenced genetically or epigenetically, which may lead to malignancy. However, research has shown that mature melanocytes may dedifferentiate, which can lead to malignancy [8].

The development of UM is primarily attributed to oxidative damage in ocular pigmented tissues. The term “ocular pigmented tissues” refers to components of the eye that are involved in pigment production (iris, ciliary body, and choroid) [9–11]. Additionally, genetic alterations, such as abnormal adenine-to-cytosine mutations in UM and adenine-to-thymine mutations in ciliochoroidal melanoma, contribute to its pathogenesis [2]. Histologically, UM manifests as spindle cell melanoma, mixed cell melanoma, or epithelioid cell melanoma [12]. It is important to note that prognosis in UM is closely associated with nuclear grade and cell type in histopathological sections. Tumors predominantly composed of epithelioid cells tend to have a poorer prognosis compared to those primarily composed of spindle-like cells, while tumors with a mixed cell type exhibit an intermediate risk [13, 14]. Spindle cell histology is more prevalent in younger patients. Notably, metastatic UM typically exhibits predominantly epithelioid cells, but spindle-like cells are always present [13]. Additionally, pathological examination often employs S-100 protein staining, which is the most sensitive for melanocytic lesions. Other melanoma-specific markers such as human melanoma black 45 (HMB45), tyrosinase, MART-1/Melan-A, melanocyte-inducing transcription factor (MITF), and sex-determining region Y-box 10 (SOX10), aid in melanoma diagnosis [12, 15].

Intense efforts in recent decades have focused on unraveling the molecular genetics underlying the development and progression of UM, particularly in identifying markers predictive of metastasis and elucidating relevant signaling pathways [16].

## Cytogenetic changes and chromosomal abnormalities

Certain chromosomal abnormalities have been linked to UM prognosis and metastatic behavior. Disomy of chromosome 3 and gain of chromosome 6p are associated with a favorable prognosis. In contrast, loss of chromosomes 1p, 3, 6q, and 8p, as well as gain of chromosome 8q are associated with higher mortality [17]. Monosomy of chromosome 3 and polysomy of chromosome 8q correlate with the involvement of the ciliary body, increased tumor basal diameter, and presence of epithelioid cells [18].

## Molecular and cellular phenomena

Several signaling pathways have been implicated in UM, with the most notable being the rat sarcoma virus/mitogen-activated protein kinases (RAS/MAPK) pathway, phosphoinositide 3-kinase (PI3K)/Akt/mammalian target of rapamycin (mTOR) pathway, Hippo-yes-activated protein (YAP) pathway, retinoblastoma (Rb) pathway, and p53 pathway.

## RAS/MAPK pathway

The RAS/MAPK pathway acts as a signaling cascade between the cell membrane and DNA inside the nucleus. This pathway has various cellular effects, such as mediating the cell cycle. Activation of this pathway leads to the proliferation of neoplastic cells in an autonomous manner. In most cases of UM, this pathway is constitutively activated due to mutations in guanine nucleotide-binding protein G(q) subunit alpha (*GNAQ*) or guanine nucleotide-binding protein subunit alpha-11 (*GNA11*). *GNAQ*/*GNA11* are members of G(q) family, and their mutation occurs in more than 80% of UM cases. The acquisition of *GNAQ* mutation occurs somatically [19, 20]. In cases of UM not caused by *GNAQ* or *GNA11* mutations, mutations in other genes within the G-alpha pathway, such as cysteinyl leukotriene receptor 2 (*CYSLTR2*) and phospholipase C beta 4 (*PLCβ4*), are often found in a mutually independent manner [21, 22].

CYSLTR2 is located at the cell surface and when activated, it leads to activation of *GNAQ* and *GNA11*, allowing the G protein to be released from the CYSLTR2 receptor. This process activates several downstream effectors, including PLCβ4 and ARF6. PLCβ4 indirectly activates MAPK and Akt/mTOR pathways, resulting in cell growth and proliferation [23, 24].

**Figure 1:**
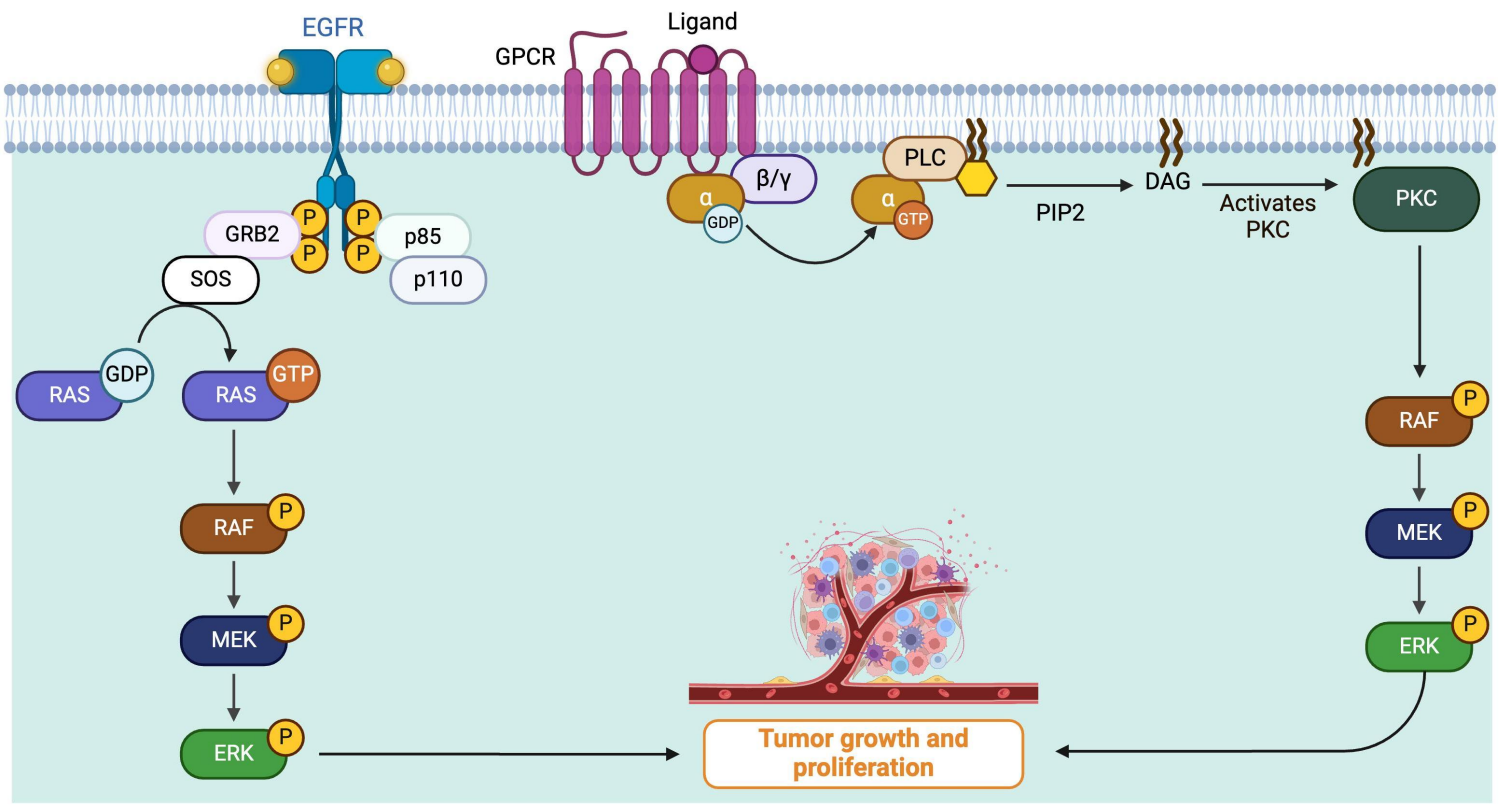
**RAS/MAPK pathway role in tumor growth and cell proliferation of uveal melanoma.** Cell surface receptors are stimulated and start intracellular pathways which finally result in cancerous cell proliferation. Growth factor binds to and activates membrane receptors, subsequently, a membrane-bound protein named RAS is activated. This leads to the phosphorylation (denoted by P) or activation of RAS which recruits RAF (ARAF/BRAF/CRAF) which leads to the activation of MEK1 and MEK2. Finally, ERK1 and ERK2 are activated causing tumor growth. Mutations in Gα (*GNAQ*/*GNA11* isoforms) induce PLCβ activation which leads to persistent MAPK cascade and finally tumor growth. DAG: diacylglycerol; EGFR: epidermal growth factor receptor; GDP: guanosine diphosphate; *GNA11*: guanine nucleotide-binding protein subunit alpha-11; *GNAQ*: guanine nucleotide-binding protein G(q) subunit alpha; GPCR: G-protein coupled receptor; GRB2: growth factor receptor-bound protein 2; GTP: guanosine triphosphate; MAPK: mitogen-activated protein kinases; PKC: protein kinase C; PLC: phospholipase C; RAS: rat sarcoma virus; RTK: receptor tyrosine kinase. Created in BioRender. Prakash, A. (2025) https://BioRender.com/f79v771

RAS serves as the mediator between cell surface receptors and MAPKs. MAPKs are part of a major signaling cascade in the cell, specific to serine or threonine amino acids (Ser/Thr protein kinases). They are responsible for communication between the stimulation of extracellular growth factors (GFs) on specific receptors and various downstream substrates. Therefore, MAPKs direct cellular responses to various stimuli. In the RAS/MAPK pathway, a ligand (e.g., a GF) binds to and activates a receptor tyrosine kinase (RTK) on the cell surface which, subsequently, activates a membrane-bound protein named RAS by raising guanine nucleotide exchange factors (GEFs) such as SOS1 and SOS2. RAS is bound to a guanosine diphosphate (GDP) and when activated, RAS swaps its GDP for a guanosine triphosphate (GTP). This initiates the kinase cascade (MAPKs). The activated RAS recruits RAF (ARAF/BRAF/CRAF), a high-activity dimer (which can be homodimer or heterodimer). The RAF dimer then phosphorylates and activates MEK1 and MEK2. Subsequently, MEK1/2 activates ERK1 and ERK2 (Figure 1) [25–27].

Mutations in *GNAQ*/*GNA11* result in activation of PLCβ, leading to increased levels of inositol 1,4,5-triphosphate (IP3) and diacylglycerol (DAG) in the cell. IP3 induces a rapid rise in Ca^2+^ levels in the cytoplasm. This increase in calcium activates various calcium-regulated pathways, while DAG causes stimulation of protein kinase C (PKC), leading to the activation of MAPK. Therefore, *GNAQ*/*GNA11* mutations can result in constitutive activation of MAP kinases (Figure 1) [28, 29].

UMs frequently exhibit mutations in Gαq, resulting in persistent activation of the MAPK pathway. While MEK inhibitors have demonstrated limited effectiveness against UM in clinical settings, combining Gαq inhibitors with MEK inhibitors has shown promise in preclinical models. This combination achieved sustained suppression of MAPK signaling and yielded enhanced therapeutic outcomes [30].

## PI3K/Akt/mTOR pathway

**Figure 2:**
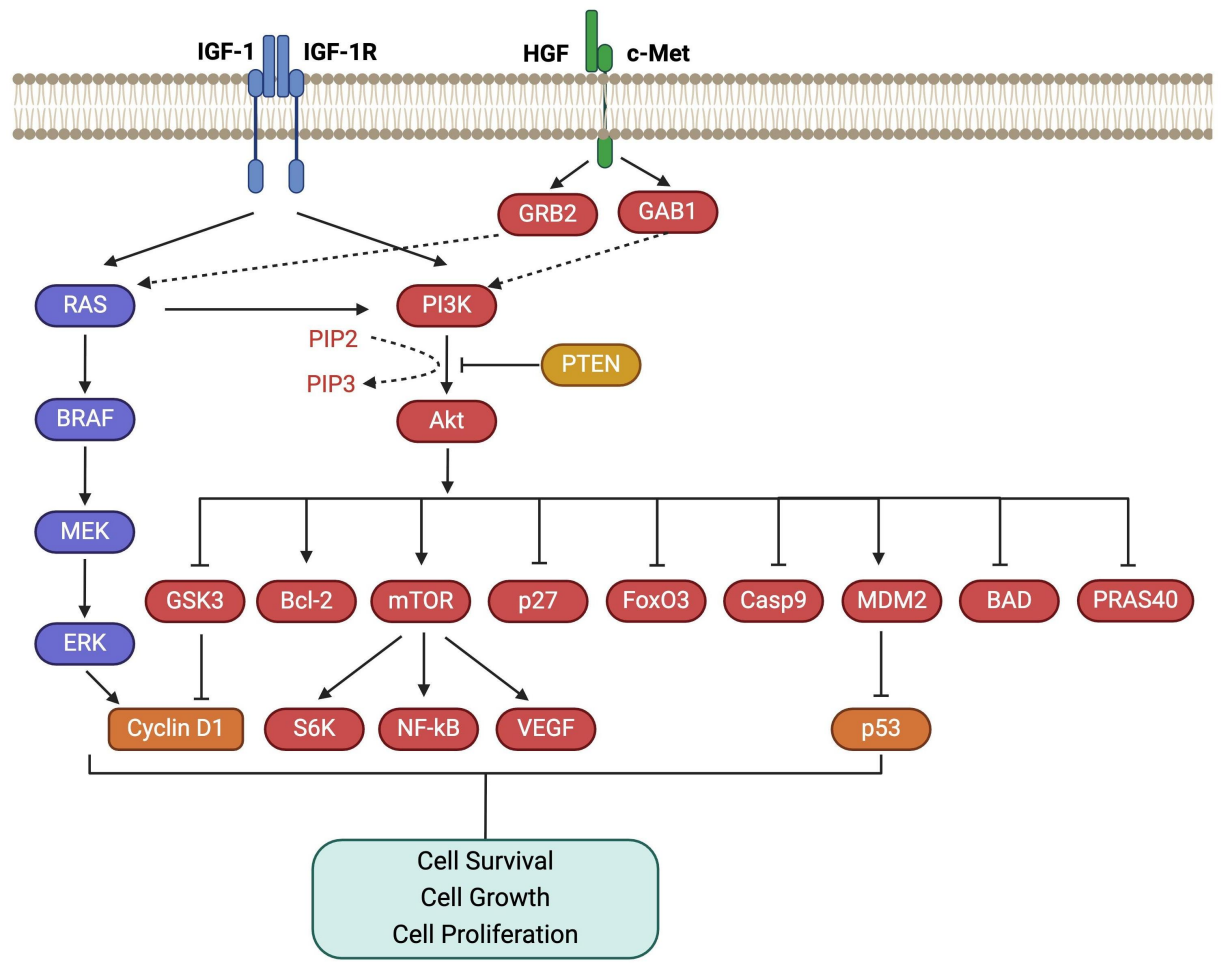
**PI3K/Akt/mTOR pathway.** The PI3K/Akt/mTOR pathway is activated by growth factors like HGF and IGF-1, promoting cell growth and tumor proliferation. GAB1: growth factor receptor-bound protein 2 associated binding protein 1; GRB2: growth factor receptor-bound protein 2; HGF: hepatocyte growth factor; IGF-1: insulin growth factor 1; IGF-1R: insulin growth factor 1 receptor; mTOR: mammalian target of rapamycin; NF-kB: nuclear factor kappa B; PI3K: phosphoinositide 3 kinase; PTEN: phosphatase and tensin homolog; RAS: rat sarcoma virus; VEGF: vascular endothelial growth factor. Created in BioRender. Prakash, A. (2025) https://BioRender.com/f79v771

The PI3K/PKB also known as Akt/mTOR pathway, is a complex intracellular pathway that drives cell growth and tumor proliferation (Figure 2). Hepatocyte GF (HGF), along with its receptor, c-Met (HGFR), and insulin GF 1 (IGF-1) with its receptor (IGF-1R), have been shown to be the activators of both RAS/MAPK and PI3K/Akt/mTOR pathways [31–33]. Activation of PI3K catalyzes the conversion of PIP2 to PIP3, which subsequently activates Akt through phosphorylation. Conversely, PTEN (phosphatase and tensin homolog) antagonizes PI3K pathway signaling by converting PIP3 back to PIP2 [34]. Phosphorylated Akt, in turn, phosphorylates its downstream targets, inhibiting GSK3, BAD, Casp9, p27, PRAS40, and FoxO3, and activating mTOR, MDM2, and Bcl-2, which promote cell survival and proliferation. mTOR is a Ser/Thr protein kinase involved in protein synthesis and MDM2 acts as an inhibitor of p53, while GSK3 suppresses cyclin D1 expression. As a result, the PI3K pathway triggers a network that positively regulates G1/S cell cycle progression and increases cyclin D1 expression [19, 31, 35–37]. PI3K/Akt pathway is also involved in the downregulation of cell adhesion molecules, including E-cadherin, and β-catenin, contributing to enhanced motility and migration of UM cells to the liver [31, 32].

Geng et al. [38] investigated pathway-related genes (PRGs) and developed a novel prognostic risk model. One of subtypes identified in this study, which had the worst survival rates, exhibited elevated human leukocyte antigen expression, immune checkpoint activity, and immune cell infiltration. Enrichment analysis linked this subtype to inflammatory responses, apoptosis, angiogenesis, and the PI3K/AKT/mTOR pathway. In this study, using 56 differentially expressed genes, a robust machine learning-based random survival forest model was created, offering high accuracy in predicting patient outcomes. This signature also revealed distinct immune statuses and mutations, suggesting potential for personalized UM therapies using PI3K/AKT/mTOR pathway [38].

## Hippo/YAP pathway

**Figure 3:**
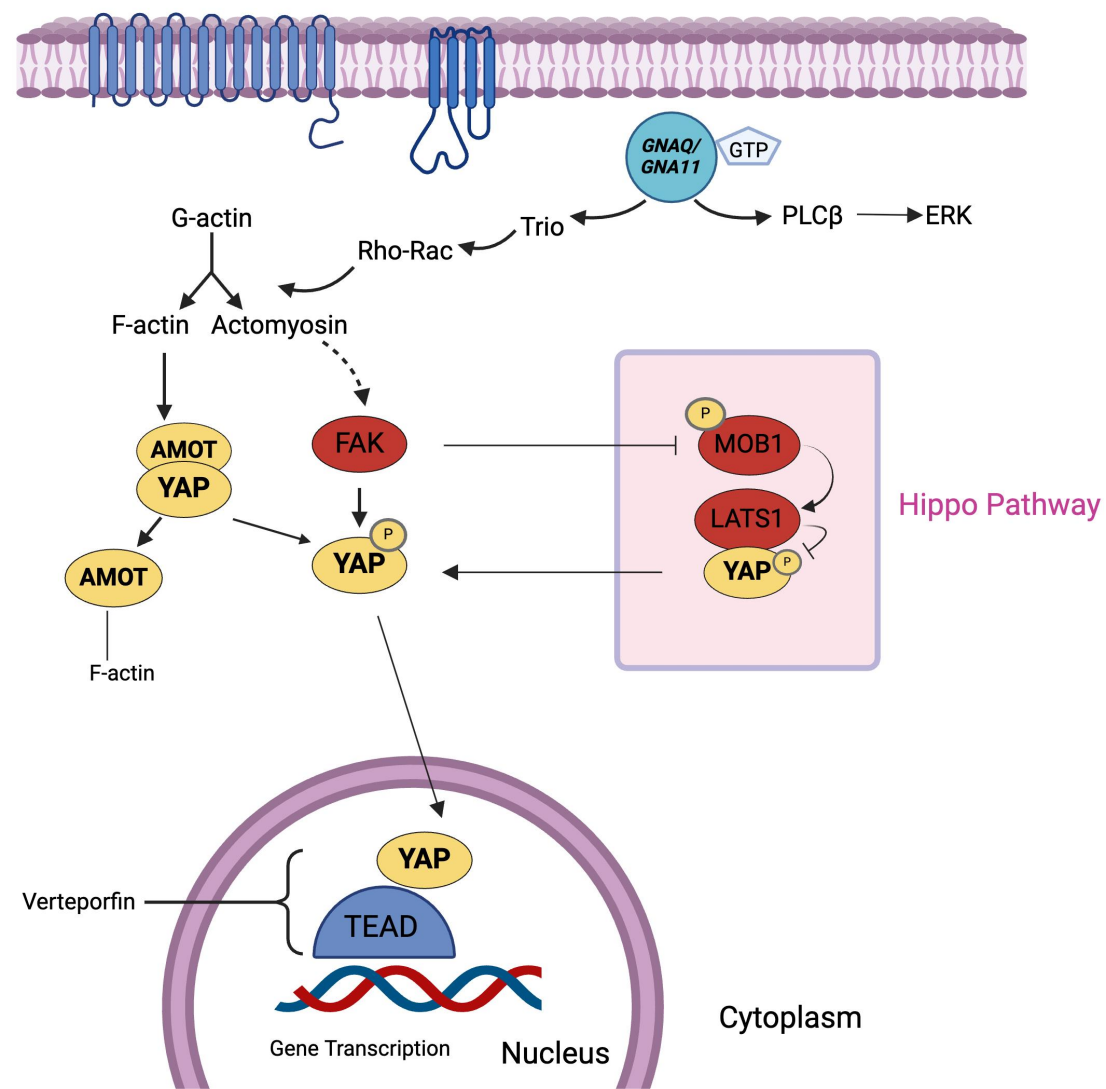
**Hippo/YAP pathway.** Hippo pathway regulates YAP by phosphorylation (denoted by P), limiting its transcriptional function. *GNAQ*/*GNA11* mutations activate the YAP pathway, promoting cell survival and proliferation, either directly or through Hippo pathway inhibition. F-actin: filamentous actin; FAK: focal adhesion kinase; G-actin: globular actin; *GNA11*: guanine nucleotide-binding protein subunit alpha-11; *GNAQ*: guanine nucleotide-binding protein G(q) subunit alpha; GTP: guanosine triphosphate; PLCβ: phospholipase C beta; YAP: yes-activated protein. Created in BioRender. Prakash, A. (2025) https://BioRender.com/e87w713

The Hippo pathway is a Ser/Thr kinase module that phosphorylates YAP, a transcriptional coactivator, on Ser residues, particularly Ser 127. This phosphorylation leads to the sequestration of YAP in the cytoplasm, thus inhibiting YAP pathway and limiting its coactivating transcriptional function (Figure 3). This pathway is also critical in the pathobiology of hepatocellular carcinoma and cholangiocarcinoma [39]. Mutation in *GNAQ* and *GNA11* can activate the YAP pathway through the Trio-RhoGTPase/RhoA/Rac1 signaling circuit, promoting the polymerization of globular actin (G-actin) to filamentous actin (F-actin) and actomyosin. F-actin binds to the cytoskeletal protein angiomotin, which leads to the release of YAP, allowing it to enter the nucleus. Meanwhile, actomyosin induces cell contraction and activates focal adhesion kinase (FAK), which, in turn, results in tyrosine phosphorylation of YAP and MOB1. MOB1, a component of the Hippo pathway, is inhibited by phosphorylation, leading to the inhibition of the Hippo pathway. Phosphorylation of YAP promotes its entry to the nucleus, where it initiates the transcription of genes such as *CTGF* and *CYR61*, which are involved in proliferation, anti-apoptosis, and cell survival. Therefore, mutations in *GNAQ*/*GNA11* can lead to the activation of the YAP pathway either directly or through inhibition of the Hippo pathway [40–42].

A study aimed to assess the relationship between YAP/TAZ activation in UM and the genetic background of tumors, along with the sensitivity of melanoma cell lines to verteporfin (VP)-mediated YAP/TAZ inhibition. Analysis of 144 enucleated UM cases, including data from The Cancer Genome Atlas (TCGA) and Leiden cohorts, revealed that high *YAP1* and *WWTR1* expression correlated with monosomy 3, BRCA1-associated protein 1 (*BAP1*) loss, and metastasis development. *TEAD4* expression was similarly associated with high-risk features. VP reduced proliferation in fast-growing UM cell lines with *BAP1*-positive or *GNAQ*/*GNA11* mutations but was ineffective in *BAP1*-negative or conjunctival melanoma cell lines. Findings suggest that genetic background and cell growth rates significantly influence the response to VP-mediated YAP/TAZ inhibition [43].

## Rb pathway

Rb is considered the prototype tumor suppressor protein. As a cell proliferation inhibitor, it induces G_1_ phase arrest in the cell cycle. In its active hypophosphorylated form, Rb binds to the E2F family of TFs, preventing the transcription of S-phase genes. When a GF signaling leads to the expression of cyclin D-cyclin-dependent kinase 4/6 (CDK4/6) complexes, Rb is inactivated by cyclin D-CDK4/6 dependent hyperphosphorylation, facilitated by small ubiquitin-like modifier (SUMO)ylation. This phosphorylation causes Rb to release E2F, allowing the cell to enter the S-phase [19, 44–47].

Disruption of Rb pathway is a common event in most cancers. It can be functionally inactivated by phosphorylation induced by upstream effectors of Rb pathway. Inactivation mechanisms for Rb protein in UM can occur due to cyclin D1 overexpression (seen in 65% of cases) or CDK-inhibitors (CDKIs) inactivation. Amplification or translocation in cyclin D gene and some other rearrangements can cause cyclin D overexpression, which activates cyclin D-CDK4/6 complexes. These complexes, in turn, phosphorylate Rb at CDK-phosphoacceptor sites, leading to Rb inactivation. This loss of Rb’s tumor-suppressing function prevents cell arrest in the G_1_ phase, ultimately promoting cancer development. In addition to cyclin D1, other regulators such as cyclin D2, cyclin D3, cyclin E, or even CD4/6 can also be dysregulated in certain cases, contributing to Rb pathway disruption [19, 46–49].

In another mechanism, cyclin D can compete directly or indirectly with CDKIs such as p16, p21, and p27. As a result, cyclin D overexpression prevents the formation and proper function of CDK-CDKI complexes, leading to Rb phosphorylation and its inactivation [19, 47].

## p53 pathway

**Figure 4:**
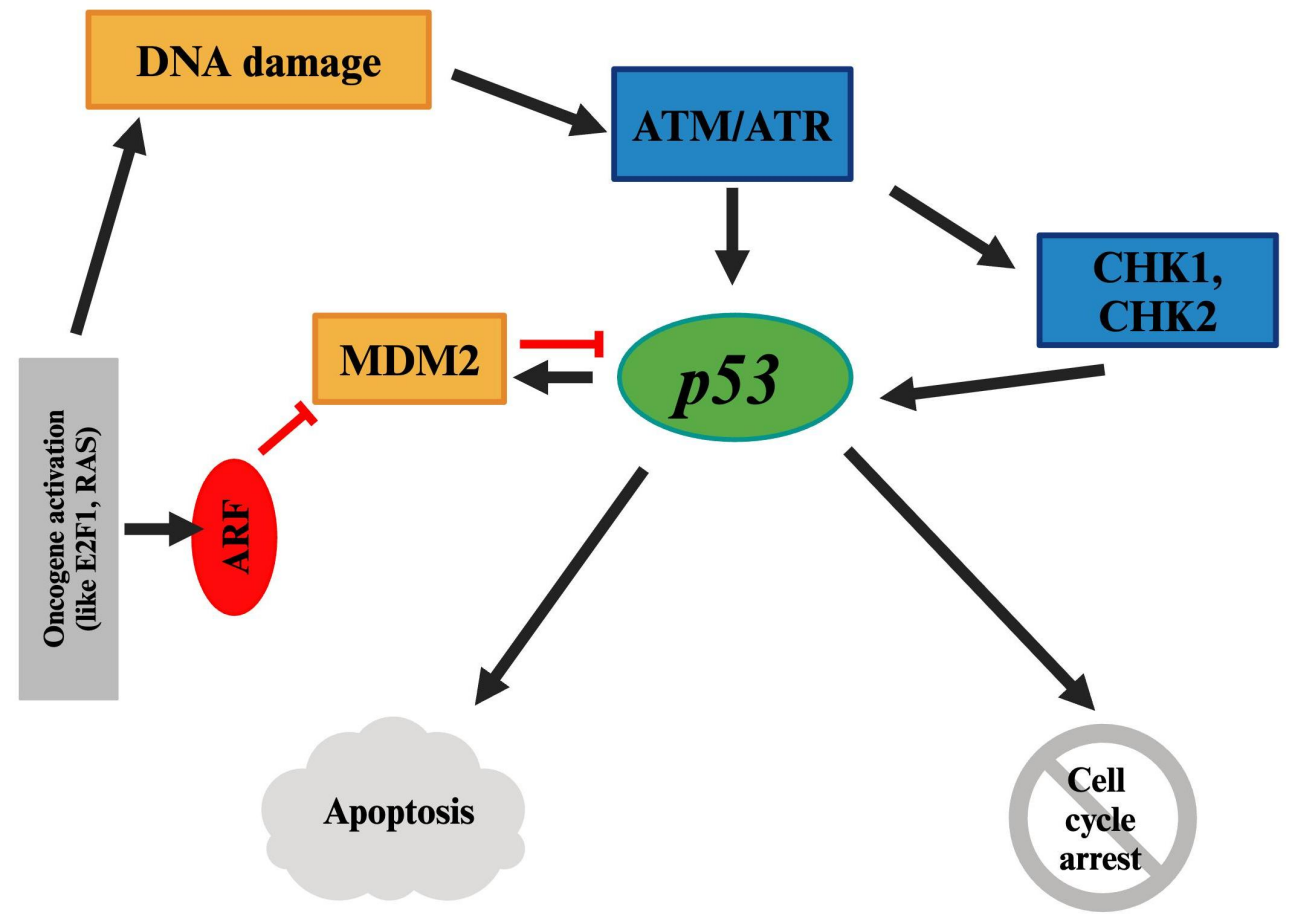
**p53 pathway.** Mutations or disruptions in the p53 pathway can lead to genetic instability and malignancy, though *TP53* mutations are rare in UM. RAS: rat sarcoma virus; UM: uveal melanoma. Created in BioRender. Prakash, A. (2025) https://BioRender.com/e87w713

p53, the most important regulator of apoptosis, is encoded by the *TP53* gene on human chromosome 17. It plays a crucial role in development, aging, and cellular homeostasis, and acts as a defense against cytogenetic or other cellular malignancies. In response to activators of p53, either apoptosis or cell cycle arrest can occur (Figure 4). Mutations in the *TP53* gene, deregulation of p53 pathway, or mechanisms that inactivate the p53, result in p53 function loss. Consequently, inefficiency of p53 allows the accumulation of genetic mutations, and p53 triggers (such as Rb loss) would no longer have an effect, all of which promote the progression toward malignancy [50–52]. Disruption of p53 is reported in over 50% of human cancers [51]. However, the *TP53* gene mutations have only been reported in rare cases of UM [19, 53–55].

*MDM2* amplification, loss of p14ARF, and mutations in activating kinases like ATM and CHK2 can inhibit p53; thus, leading to the inhibition of apoptosis following cellular and genetic damages [19, 51]. High expression of p53 in UM has been reported to be correlated with poor prognosis [19, 54, 55].

Bcl-2, which is directly regulated by p53, is a critical anti-apoptotic protein. Its deregulation is one of the most common molecular abnormalities, and it has been reported to be overexpressed in UM [19, 56–58].

PI3K/Akt/mTOR pathways are interrelated through Akt, which upregulates MDM2, leading to inhibition of p53 [31]. Additionally, p53 and Rb pathways are interconnected via the ARF-MDM2 axis. Activation of oncogenes such as E2F can induce ARF expression and activation, which directly antagonizes MDM2, allowing p53 to accumulate. Ultimately, p53 can induce apoptosis [45].

## Hallmarks of cancer

Hallmarks of cancer are relevant to UM; however, further research and studies are needed. For instance, the cell cycle is disrupted by the overexpression of cyclin D1, Rb protein hyperphosphorylation (resulting in functional inactivation), and inactivation of the *INK4A* (*p16*) gene. Functional inhibition in Rb protein and p53 pathway is more common than hypermethylation of *RB1* or *TP53* genes [44, 47, 59]. Consequently, the cell cycle loses its regulatory inhibition. Suppression of cell cycle inhibitors like Rb and p53 can lead to malignant changes. These changes contribute to the first hallmark of cancer, insensitivity to anti-growth signals [60]. In addition to the deactivation or disruption of inhibitory pathways, there are also activated pathways. Activating mutations in *GNAQ* or *GNA11* (which occur in more than 80% of UM cases) can constitutively activate proteins and downstream signaling pathways such as RAF/MEK/ERK, YAP, and PI3K/Akt/mTOR pathways. These activated pathways lead to self-sufficiency in GFs, the second hallmark of cancer [19].

Metastasis and dissemination of cancerous cells are influenced by several signaling pathways, including *LZTS1* (a tumor suppressor gene located on chromosome 8p) [61], *DDEF1* [also known as *ASAP1* (a proto-oncogene)] [62], *PTP4A3* (protein tyrosine phosphatase type IVA member 3) [63], *TCEB1* (transcription elongation factor B polypeptide 1), and *NOTCH* signaling [64]. The liver is the most common site of metastasis, occurring in 90% of cases of UM cases with metastasis. At the time of death, 50% of UM cases have been reported to have metastasis confined to the liver. Despite treatment, metastasis occurs in up to 50% of UM cases. The prognosis for metastatic UM is poor, with a one-year survival rate of approximately 15%, and a median survival period from 4 to 15 months [65]. However, only 4% of UM cases exhibit detectable metastasis at the time of diagnosis [66].

High vascularization is a key characteristic of UM. The vascular endothelial GF (VEGF) and its receptor (VEGFR) pathway play a significant role in UM tumor vascularization. Both stromal and cancerous cells secrete VEGF. Interleukin-8 (IL-8), also known as CXCL8, is frequently expressed alongside VEGF. IL-8 can affect endothelial cells, leading to the expression of VEGFR2 and VEGF-A, which promote angiogenesis. IL-8 and VEGF cooperate and synergistically reciprocally upregulate angiogenesis. Two important G protein-coupled receptors on the cell surface that mediate the effects of IL-8, are CXCR1 and CXCR2 [67–74]. CXCR1 is activated solely by IL-8; while, CXCR2 can bind to some different CXC chemokines, including melanoma growth stimulatory activity (MGSA) [75]. Overexpression of IL-8 is commonly observed in numerous tumors due to therapies (e.g., chemotherapy and radiotherapy) or hypoxia. Therefore, IL-8 is implicated in tumor progression [76–78]. Overall, IL-8 concentration has been shown to be elevated in ocular fluids of UM cases [49].

Generally, it has been observed that UM tumors with monosomy of chromosome 3 exhibit increased HLA expression and a higher presence of M2-type macrophages compared to their chromosome 3 disomy counterparts. An inflammatory phenotype in UM has been linked to monosomy of chromosome 3 and is associated with poor prognosis. These tumors predominantly contain M2-type tumor-associated macrophages (TAMs) which contribute to cell proliferation via the GFs production and promote angiogenesis via VEGF secretion, in addition to their immunosuppressive properties [79–81].

It is noteworthy to mention that melanocytes, derived from embryonic neural crest cells (NCCs), are melanin-producing cells found in the epidermis, hair, and iris, as well as in the nervous system, inner ear, and heart. Other melanin-producing cells include those in the retinal pigmented epithelium, iris and ciliary body epithelia, adipocytes, and some neurons. The primary inducer of melanogenesis is α-melanocyte-stimulating hormone (α-MSH), which acts via the melanocortin receptor 1 (MC1R). Tyrosinase, aided by tyrosinase-related proteins 1 and 2, catalyzes melanin biosynthesis, with MITF as the key regulatory TF. MITF expression is modulated by signaling pathways such as PI3K/Akt, which suppresses its expression, and ERK, which promotes its degradation. While cutaneous melanogenesis has been extensively studied, the regulation of uveal melanocytes remains poorly understood. Notably, prostaglandin analogs used for glaucoma treatment can induce pigmentation changes in periocular skin and iris, suggesting some regulatory capacity in adult eye melanogenesis [82].

## Genetic and epigenetic alterations

Five key genes—*BAP1*, *EIF1AX*, *GNA11*, *GNAQ*, *SF3B1*—have been identified as more frequently mutated in UM compared to other genes. These mutations are categorized based on their timing during tumor development, with early-onset mutations (*GNA11*, *GNAQ*) and late-onset mutations (*BAP1*, *EIF1AX*, *SF3B1*). *GNAQ* and *GNA11* mutations are the primary initiators of UM development and are not prognostically significant. Two additional genes, *CYSLTR2* and *PLCβ4*, also have early-onset mutations in UM, though they are less frequently mutated. On the other hand, *BAP1*, *SF3B1*, and *EIF1AX* mutations occur during UM progression in a mutually independent manner and have varying levels of metastatic risk, making them useful as a prognostic factor [20–22, 31, 83]. *BAP1* mutation, present in nearly half of all UM cases, is associated with a high metastatic risk (typically leading to metastasis within 5 years) [84]. In contrast, *SF3B1* mutation carries an intermediate metastatic risk (mostly resulting in metastasis within 15 years) [85], while the *EIF1AX* mutation is associated with a low metastatic risk (rarely leading to metastasis) [83]. Most *BAP1*-mutated UM cases exhibit loss of chromosome 3 and gain of 8q, which are chromosomal changes associated with bad prognosis. In contrast, tumors with a *EIF1AX* or *SF3B1* mutations frequently show a gain of chromosome 6p, a chromosomal change associated with favorable prognosis [86].

*NBS1* (Nijmegen breakage syndrome 1) gene amplification occurs in 50% in UM cases. This gene is involved in the repair of double-strand DNA damage [87]. *CCND1*, which encodes cyclin D1, is amplified in 65% of UM cases. *Bcl-2* amplification, with a frequency exceeding 95%, is one of the most important genetic alterations in UM [88, 89]. Additionally, *RASSF1* (RAS association domain family protein 1A) hypermethylation, which occurs in 13–70% of UM cases, is an important epigenetic alteration in UM [90].

Although there are some similarities in pathobiology and molecular events between cutaneous and UM, there are also major differences. For example, while *BRAF* mutations serve as initiators of malignancy in cutaneous melanoma, *GNAQ* and *GNA11* mutations act as initiators in UM. However, the mutations of *GNAQ* and *GNA11* are not sufficient on their own to fully transform cells into malignant cells. Nevertheless, they play a crucial role in the early stages of molecular pathology of UM [27].

*BAP1* is a tumor suppressor gene located on chromosome 3p21 and is mutated in 47% of primary UM. It plays a critical role in maintaining genome stability, cell identity, and regulating the cell cycle. *BAP1* mutations typically occur following *GNAQ* and *GNA11* mutations [91]. These mutations are strongly associated with a high likelihood of metastasis in UM. One notable alteration is in the ubiquitin carboxy-terminal hydrolase (UCH) domain, where loss of UCH activity predisposes cells to metastasis. Host cell factor-1 and histone H2A are some of the key targets of *BAP1* UCH activity in UM. A reduction of *BAP1* activity causes UM cells to acquire characteristics reminiscent of stem cells, leading to upregulation of genes with stem cell-like properties and developmental processes. Meanwhile, the transcriptional program of melanocytes is downregulated, and morphological features of cellular differentiation, such as dendritic arborizations and spindle morphology are diminished [84, 92–95].

Epigenetic factors include the regulation of microRNAs (miRNAs) and promotors methylation of certain genes. miRNAs are small, non-coding, single-stranded RNA molecules that can regulate gene expression. Several miRNAs have been implicated in UM development. Downregulation of miR-182 (a p53-dependent miRNA), miR-145 and miR-204, and upregulation of let-7b, miR-199a, miR-143, miR-193b, miR-20a, miR-106a, miR-17, miR-21, miR-34a, and miR-181b have been observed in UM. Overexpression of miR-181b inhibits CTD small phosphatase-like protein (*CTDSPL*) expression, leading to phosphorylation of Rb and can play a pivotal role in the development of UM [96–102]. Additionally, miRNA-145 suppresses UM angiogenesis by inhibiting the N-RAS/VEGF signaling pathway [103].

Promoter methylation is another important epigenetic factor in play. In UM, increased promotor methylation in the tumor suppressor genes such as multiple EGF-like domains 10 (*MEGF10*), Glutathione S-transferase Pi 1 (*GSTP1*), and kruppel-like factor 10 (*KLF10*) has been observed. *MEGF10* is a gene that regulates cell migration and adhesion, while *GSTP1* regulates oncogenic signaling pathways by activating glyceraldehyde-3-phosphate dehydrogenase [104–106]. *KLF10* is a DNA-transcription regulator that binds to GC-rich sequences in gene promoters to inhibit growth and initiate apoptosis through transforming GF beta (TGF-β) signaling [104, 107].

Altogether, genetic and molecular analyses can effectively identify high-risk patients, guide follow-up strategies, and aid in the development of targeted therapy by addressing key signaling pathways. Unique miRNA expression profiles in melanoma tissue offer potential diagnostic and prognostic applications. Although liver function tests are not definitive for detecting liver metastases, they can serve as supplementary indicators. Epigenetic dysregulation, including DNA methylation and histone modifications, plays a crucial role in UM, underscoring the potential of combination therapies with advanced epigenetic drugs. Emerging technologies like CRISPR-dCas9 hold significant promise for uncovering and targeting driver epigenetic changes, paving the way for innovative treatments [108, 109].

It is noteworthy to mention that vitamin D3 signaling role in UM. Vitamin D3 is synthesized in the skin via UVB exposure, fulfilling over 90% of the body’s requirement, and is activated through hydroxylation by enzymes like CYP2R1, CYP27A1, and CYP27B1 to produce bioactive forms such as 1,25(OH)_2_D3. These active forms regulate calcium metabolism and exhibit anticarcinogenic, anti-melanoma, and photoprotective effects, primarily via interactions with the vitamin D receptor (VDR) and retinoic acid orphan receptors (ROR)α and RORγ. Low levels of 25(OH)D correlate with worse melanoma outcomes, including thicker tumors and reduced survival. Genetic variations in VDR and the vitamin D-binding protein (*VDP*) genes also influence melanoma progression. Low or absent VDR and CYP27B1 expression is linked to shorter overall survival, while high CYP24A1 levels, which inactivate 1,25(OH)_2_D3 but activate alternative D3 forms, paradoxically indicate better outcomes. These findings highlight the complex roles of D3 forms and receptors in melanoma and the importance of considering them in therapeutic strategies [110].

Altogether, it should be noted that advanced tumors including melanoma can autoregulate not only its environment but also body homeostasis.

## Conclusions

The aim of this study was to provide an overview of the latest insights into the pathogenesis of uveal melanoma (UM). As outlined in this article, the multifactorial nature of UM becomes evident through the alterations in intracellular pathways, the activation or inactivation of specific molecules and enzymes, and the altered expression of UM-related genes and their downstream effects. Additionally, the metastasis of UM and its involvement in various organs, along with chromosomal changes, underscore the complexity of this cancer. These findings highlight the need for further research to fully elucidate the unknown aspects of UM pathogenesis. Such studies are critical for developing targeted therapies that can prevent treatment failure and disease relapse. A key knowledge gap remains in identifying the most effective targeted therapies and understanding how different pathways are interconnected in the progression of UM.

## Abbreviations

*BAP1*: BRCA1-associated protein 1
CDK4/6: cyclin-dependent kinase 4/6
CDKIs: cyclin-dependent kinase inhibitors
*CYSLTR2*: cysteinyl leukotriene receptor 2
DAG: diacylglycerol
F-actin: filamentous actin
GDP: guanosine diphosphate
GFs: growth factors
*GNA11*: guanine nucleotide-binding protein subunit alpha-11
*GNAQ*: guanine nucleotide-binding protein G(q) subunit alpha
*GSTP1*: Glutathione S-transferase Pi 1
IL-8: interleukin-8
IP3: inositol 1,4,5-triphosphate
*KLF10*: kruppel-like factor 10
MAPK: mitogen-activated protein kinases
*MEGF10*: multiple EGF-like domains 10
miRNAs: microRNAs
MITF: melanocyte-inducing transcription factor
mTOR: mammalian target of rapamycin
PI3K: phosphoinositide 3 kinase
*PLCβ4*: phospholipase C beta 4
RAS: rat sarcoma virus
Rb: retinoblastoma
ROR: retinoic acid orphan receptors
Ser: serine
TFs: transcription factors
Thr: threonine
UCH: ubiquitin carboxy-terminal hydrolase
UM: uveal melanoma
VDR: vitamin D receptor
VEGF: vascular endothelial growth factor
VEGFR: vascular endothelial growth factor receptor
VP: verteporfin
YAP: yes-activated protein

## Declarations

### Copyright

© The Author(s) 2025.
